# PReS-FINAL-2090: Efficacy of anti-TNF drugs from the perspective of growth velocity: a single center experience

**DOI:** 10.1186/1546-0096-11-S2-P102

**Published:** 2013-12-05

**Authors:** P Mrážik, V Vargová

**Affiliations:** 1 1st Department of Paediatrics and Adolescent Medicine, Šafárik University and Children Faculty Hospital in Košice, Košice, Slovakia

## Introduction

Growth is a sensitive marker of well-being in children. In patients with juvenile idiopathic arthritis (JIA) both, ongoing inflammation and corticosteroid treatment, may affect linear growth adversely, potentially leading to irreversible growth retardation. Biologic treatment of JIA may have the potential to enable normal growth in children with JIA.

## Objectives

To retrospectively evaluate the safety, clinical efficacy, corticosteroid-dose and impact on linear growth of biologics in children with JIA in The center of the biological treatment for children with chronic rheumatic diseases in Košice.

## Methods

Inclusion criteria:

- DMARDs-resistant polyarticular course of JIA receiving anti-TNF treatment at least one year.

- Evaluation of response every 3 months by Pediatric American College of Rheumatology Response Criteria (ACR Paed).

Exclusion criteria:

- no growth expected boys: Tanner staging sexual maturity score V plus age 17 before starting anti-TNF treatment

- no growth expected girls: menarche at least two years before starting anti-TNF treatment or menarche at least a year before starting treatment plus Tanner staging sexual maturity score IV

Growth velocity was defined by change of height in standard deviation (SD) score. To calculate exact SD score software Rust CZ 2.0, based on anthropometric data of Czech paediatric population - the nearest one to Slovak paediatric population, was used. Catch-up was defined as positive growth velocity.

## Results

The group of 20 patients with expected growth (boys vs. girls: 10 vs. 10; etanercept vs. adalimumab: 16 vs. 4) was assessed. The mean age at anti-TNF treatment initiation was 12.03 years (SD ± 4.44). Median duration of disease was 3.25 years (range 0.89-10.23), 15 patients received corticosteroids in mean dose 0.273 mg/kg/day.

All patients were identified as "responders" (ACR Paed 30) at 3 months. One patient was a ...secondary non-responder" at 6 months, but completed 12 months of treatment. The daily dose of corticosteroids at 12 months was significantly reduced to 0.07 mg/kg/day (p < 0.001) and could be stopped in 10 of 15 patients. Growth catch-up was observed in 16 of 20 patients with median growth velocity being 0.325 (range -0.2 to 0.94). Two patients did not show growth during the first year of treatment.

## Conclusion

Anti-TNF treatment of JIA is highly effective and has a rapid corticosteroid-sparing effect in DMARDs-resistant JIA with polyarticular course. The clinical response is accompanied by an increase in growth rate (catch-up) in most cases.

## Disclosure of interest

None declared.

